# The State of the Art on Blood MicroRNAs in Pancreatic Ductal Adenocarcinoma

**DOI:** 10.1155/2019/9419072

**Published:** 2019-09-10

**Authors:** Zhuqing Gao, Wei Jiang, Shutian Zhang, Peng Li

**Affiliations:** ^1^Department of Gastroenterology, Beijing Friendship Hospital, Capital Medical University, Beijing 100050, China; ^2^Beijing Key Laboratory for Precancerous Lesion of Digestive Diseases, Beijing 100050, China; ^3^National Clinical Research Center for Digestive Diseases, Beijing 100050, China

## Abstract

Despite enormous advances being made in diagnosis and therapeutic interventions, pancreatic ductal adenocarcinoma (PDAC) is still recognized as one of the most lethal malignancies. Early diagnosis and timely curative surgery can markedly improve the prognosis; hence, there is an unmet necessity to explore efficient biomarkers for patients' benefit. Recently, blood miRNAs (miRNAs) have been reported to be a novel biomarker in human cancers. Part of it is selectively packaged by plasma exosomes released from cells via exocytosis and is highly sensitive to changes in the tumor microenvironment. Furthermore, due to less invasiveness and technical availability, miRNA-based liquid biopsy holds promise for further wide usage. Therefore, this review is aimed at presenting an update on the association between blood miRNAs and the biology of PDAC, then discussing its clinical utilization further.

## 1. Introduction

Pancreatic ductal adenocarcinoma (PDAC) remains the fourth leading cause of cancer-related death worldwide [1]. It has extremely poor prognosis characterized by only 18% one-year survival rate for all stages, partly due to its aggressive tumor biology such as intrinsic chemoresistance and high metastatic capacity. The lack of alarming symptoms in the early phase of PDAC makes the early diagnosis difficult; thus, only 20% of patients are suitable for potentially curative surgical resection [2]. Even when treated with surgery, the five-year survival rate for patients with node-negative and node-positive can only reach 25-30% and 10%, respectively [3]. For the majority of PDAC patients, current first-line therapy such as chemotherapy fails to improve the prognosis significantly [4]. Considering the disappointing diagnostic approaches and the poor prognosis of PDAC, it is necessary to develop tumor markers for screening, postoperative surveillance, and predicting the prognosis of curative resection. However, no biomarkers in routine practice have proven to be a powerful and widely accepted approach for large-scale screening and surveillance. For instance, carcinoembryonic antigen (CEA) and carbohydrate antigen 19-9 (CA-199) as traditional biomarkers are neither sensitive nor specific for early screening and predicting prognosis [5, 6]. The higher level of those biomarkers indicate the occurrence not only of PDAC but also of other malignancies such as colorectal carcinoma and benign lesions like pancreatitis, cirrhosis, and choledocholithiasis [7]. Moreover, many commercial kits available have not reached a widely accepted standard, and its fluctuation based on the bilirubin level limited its usage as a stand-alone test for screening and predicting prognosis [8, 9].

miRNAs have been accumulating for years [10]. They belong to a class of small noncoding RNAs (18-25 nucleotides) and can stabilize messenger RNA (mRNA) transcripts by inhibiting the translation process or cleaving their target mRNA [10–13]. While only covering 3% in the human genome, they can affect the expression level of 20-30% protein-coding genes [14, 15], which play a pivotal role in cell growth, differentiation, and apoptosis. In recent decades, the altered expression of miRNAs has been reported to have a carcinogenic or tumor-suppressing role in malignancies. The aberrant expression of miRNAs can be informative and be an indirect predictor in the development of differentiated solid tumors. Moreover, evidence showed that a substantial proportion of miRNAs abnormally expressed in PDAC. Thus, many studies were carried out to investigate whether this type of biomarkers can be a potential quantified tool for early diagnosis and prognosis prediction. miRNAs can remain stable in form and can be quantified in tissues, plasma, stool, pancreatic juice, and other fluids. Among these, the blood assay of miRNAs could potentially be an efficient regular test for the benefit of high-risk individuals, due to its less invasiveness and technology availability.

This review is aimed at presenting the evidence of blood miRNAs on early diagnosis, predictive treatment, and confirmation of postoperative prognosis in PDAC patients.

## 2. miRNAs in the Blood

Accumulating studies have reported the miRNA expression in the whole blood or peripheral blood mononuclear cell (PBMCs) [16–31] (Table 1). A study comparing the miRNA expression between healthy subjects and PDAC patients demonstrated that levels of miRNA-10b, miRNA-21, miRNA-30c, and miRNA-181a were significantly higher, whereas the expression of miRNA-let7a was lower in PDAC patients compared with controls [32]. In another study, the lower level of miRNA-155 and miRNA-196a and the increased expression of miRNA-17-5p and miRNA-21 could be observed in the subsets of PDAC patients. Interestingly, altered expression of miRNA-17-5p was linked to tumor metastasis, which could significantly affect the prognosis of patients [33].

Many investigators have formulated scenarios to explore how miRNAs can survive from endogenous ribonucleases in the blood. Currently, it is widely accepted that miRNAs can circulate in blood selectively packaged by lipoprotein vesicles, such as exosomes [34–36]. As extracellular vesicles, exosomes (30-150 nm) are secreted by all living cells via exocytosis. The exact function and mechanism of exosomes remained unknown. The current hypothesis uncovered that it may function to expel excess and nonfunctional cellular constituents. In addition, exosomes would recycle cell surface protein and modulate signaling [37, 38]. They contain the tissue-specific protein that can package RNA (mRNA and miRNA), then transport to other cells in circulating fluid. A previous study reported that the concentration of exosomes would be much higher in the blood of cancer patients than in their healthy counterparts [39]. It was also confirmed that exosome-encapsulated miRNAs can be detected in the serum of PDAC patients. The levels of miRNA-1246, miRNA-4644, miRNA-3976, and miRNA-4306 in PDAC were markedly upregulated in 83% of serum exosomes compared to control groups [40]. Another study reported that elevated levels of exosome-encapsulated miRNA-10b, miRNA-21, miRNA-30c, and miRNA-181a and decreased miRNA-let7a readily differentiate PDAC from normal control and chronic pancreatitis samples. Besides, elevated exosomal miRNA levels decreased after PDAC resection [34]. These studies suggest that serum-derived exosome miRNAs might be potential candidate biomarkers for patients with early-stage PDAC [34, 40–44] (Table 2).

## 3. Blood miRNAs in Diagnosis of PDAC

Early diagnosis and timely surgery have been reported to elicit better prognosis that the 5-year survival rate can reach 50% for PDAC in stage I [45, 46]. However, it remains an elusive goal, considering that laboratory findings did not show efficiency or high sensitivity during routine practice. Enormous endeavors have been devoted to investigating the association between miRNA expression and the diagnosis of malignancies. The goal for screening high-risk individuals has also contributed to identifying promising biomarkers for PDAC.

Tissue miRNAs have showed good performance in evaluating prognosis and survival after tumor resection. However, the invasiveness in collecting tissues and the lack of available samples have limited its usage. Inconsistent with tissues, circulating miRNAs presenting in the serum, plasma, or PBMCs can be isolated directly with minimal invasion. They are also technically easier to detect, more abundant, and resistant to the degradation of RNase. Attempts to assess the efficiency of blood miRNAs solely or in conjunction with CA19-9 have yielded various results [20, 24, 26, 47–53]. According to a study performed by Li et al., serum levels of miRNA-200a/200b were quantified in a series of 45 PDAC patients, 11 chronic pancreatitis patients, and 32 healthy counterparts. It can be observed that the elevation of miRNA-200a and miRNA-200b had a sensitivity of 84.4% and 71.1% and a specificity of 87.5% and 96.9%, respectively [53]. miRNA-16a and miRNA-196a combined with CA19-9 showed a promising result in the detection of PDAC in stage I, indicating that miRNA-16a and miRNA-196a might be used for peripheral biomarkers of PDAC [20]. Recently, a large-scale clinical trial recruiting 197 PDAC cases and 158 controls has been conducted on a miRNA panel including miRNA-20a, miRNA-21, miRNA-24, miRNA-25, miRNA-99a, miRNA-185, and miRNA-191. The overall accuracy was 86.8% compared with 76% for CA19-9. Of the stage I PDAC patients, the specificity of this seven-miRNA panel was 96.2% compared with CA199 46.2%. This panel was observed to be a potential biomarker of early diagnosis and distinguishing PDAC from chronic pancreatitis. Moreover, miRNA-1290 was also observed to distinguish early-stage PDAC patients from the targeted population [54]. The expression of miRNAs has also been evaluated in PDAC, intraductal papillary mucinous neoplasm (IPMN), and healthy subjects. In a study including 32 patients with PDAC, 12 patients with IPMN, and 30 healthy controls, the expression levels of plasma miRNA-483-3p and miRNA-21 in PDAC especially in advanced metastatic cases were significantly higher than that of healthy controls. Interestingly, the expression of plasma miRNA-483-3p in PDAC patients was also significantly higher than that in IPMN patients, which could be used to distinguish PDAC and IPMN [55].

These research focusing on detecting the role of blood miRNA in the diagnosis of PDAC has added to the evidence that it may be efficiently used for screening in routine practice in the future.

## 4. miRNA in the Treatment of PDAC

For the majority of patients in the advanced stage, chemotherapy may be the only therapy with palliative intent. However, high resistance to chemotherapy partially contributed to the poor prognosis. To date, it is accepted that PDAC cells that survived the initial chemotherapy can harbor a secondary generation of cells that can be resistant to therapy. Some studies have reported that the expression of specific miRNAs may affect characteristics of cancer stem cells. miRNA-21 has been investigated to be a potential marker of poor prognosis in PDAC. Its expression was positively correlated with the IC50 of gemcitabine. The overexpression of miRNA-21 induced by transfection was associated with enhancing proliferation, invasion, and suppressing apoptosis in PDAC cell lines [16]. Also, pancreatic cancer cells with lower miRNA-21 expression can confer higher sensitivity to 5-fluorouracil. Lately, Zhu et al. showed that transfection with miRNA-21 could increase the expression of PTEN and enhance the effect of the gemcitabine-induced cell apoptosis [56]. Moreover, the correlation between miRNA-21 and matrix metalloproteinase-2/9 as well as vascular endothelial growth factor (VEGF) was assessed in another study. A postulation can be made that miRNA-21 may also play a role in angiogenesis [57]. A further large-scale clinical trial could be carried out in a targeted population with a tendency for gemcitabine-resistance.

Apart from miRNA-21, other miRNAs have also been observed to be potential indicators in therapy. miRNA-200 was reported to be a promising tumor suppressor which plays a pivotal part in cancer metastases. It was also involved in chemoresistance. It was found that supplementation with curcumin, a dominant component of Indian spice, could upregulate miRNA-200 and downregulate miRNA-21 [58]. In another study by Zhang et al., miRNA-214 was reported to downregulate ING4 to promote the survival of PDAC cells in gemcitabine-resistance. Also, miRNA-15a can suppress the production of tumor cells in PDAC cells. Moreover, a high level of miRNA-15a inhibiting WNT3a and FGF7 expression correlates with the reduced PDAC cell viability [59].

Recently, the efficiency of nanoformulations, in which nanoparticles are combined with miRNA, has been evaluated both in the PDAC cell culture or in animal models, throwing new light on miRNA treatment in pancreatic cancer. In a study by Arora et al. [60], the level of miRNA-150 was lower in the majority of pancreatic cancer patients. Thus, poly(D, L-lactide-co-glycolide)- (PLGA-) based nanoformulations of miRNA-150 (miRNA-150-NF) were developed. Treatment with miRNA-150-NF efficiently promoted the production of the miRNA-mimic in PDAC cells and significantly downregulated the expression of its target gene, MUC4. The inhibition of MUC4 further suppressed the production of its interacting partner HER2 and inhibited its downstream signaling. Finally, the proliferation of PDAC cells was observed to be significantly inhibited. In another study, the researchers delivered the established system with two miRNAs (miRNA-34a and miRNA-143/145) encapsulated to the subcutaneous transplanted tumor cells of mice by intravenous injection. It was observed that the system can enhance the apoptosis of tumor cells and suppressed the cell proliferation. miRNA-34a is a component of the p53 transcriptional network and regulates the survival of cancer stem cells. miRNA-143/145 inhibits the expression of Kirsten rat sarcoma viral oncogene (KRAS2) and its downstream effector Ras-responsive element binding protein-1 (RREB1) [61]. Both of those miRNAs could contribute to the tumor-suppressing effect of the established system [61].

The endeavors have always been contributing to the miRNA therapy for PDAC in these years. It has shown promising results both in vivo and in vitro. In the future, its efficiency in PDAC patients needs to be validated in preclinical studies.

## 5. miRNA in the Prognosis of PDAC

As a malignancy harboring poor prognosis, it is pivotal to predict its activity when considering more precise therapy. Recently, many specific miRNA patterns have been reported to provide evidence for worse prognosis and more aggressive activity. In an analysis that enrolled 1525 patients, higher expression of miRNA-21 was observed to be correlated with a shorter significant disease-free survival. Similarly, upregulation of miRNA-155, miRNA-203, miRNA-222, and miRNA-10b and downregulation of miRNA-34a and miRNA-183 showed a worse prognosis and correlated with tumor grade, stage, and metastasis. Aberrant expression of these panels are independent predictors of worse prognosis of PDAC patients [62]. Another recent study found that high expression of miRNA-142-5p and miRNA-204 correlated with better survival. Collectively, these studies proved miRNAs as a novel biomarker for predicting prognosis [23].

Moreover, the association between 494 miRNAs and overall survival was analyzed by the Cancer Genome Atlas (TCGA). Five miRNAs significantly correlated with overall survival were miRNA-1301, miRNA-125a, miRNA-376c, miRNA-328, and miRNA-376b, which can be used as independent prognostic factors for PDAC [63]. In a study that recruited 104 patients with PDAC, three subtypes of PDAC associated with prognosis were identified by microarray analysis of 1733 miRNA expression profiles. Among 19 characteristic miRNAs, miRNA-106b-star, miRNA-324-3p, and miRNA-615 were associated with the p53 classical pathway, while miRNA-324, miRNA-145-5p, miRNA-26b-5p, and miRNA-574-3p were associated with the Cox-2 central pathway [64]. The recurrence of malignancies might be also assessed by miRNAs. Morimura et.al found that the high serum level of miRNA-18a in PDAC patients was cut off after resection of the tumors. In one case of recurrence after resection, miRNA-18a was found to be elevated. Such biomarkers can be investigated further to screen recurring tumors.

## 6. Conclusion

Current tumor biomarkers (CA19-9, CEA) in clinical practice have improved the screening for PDAC. However, this kind of implementation shows low sensitivity and specificity in some circumstances. As a novel molecular marker, blood miRNAs not only have the advantages of noninvasiveness and higher accuracy but also improve the evaluation of tumor classification, metastasis, curative effect, and recurrence. However, it is an urgent problem to find a detection method with high sensitivity, technically easier access, and lower cost due to the low expression level of miRNA in serum. What is more, a sole miRNA is often insufficiently specific. Thus, it is expected to significantly improve the accuracy of the diagnosis if a panel of miRNAs can be used. As an emerging tumor molecular marker category, blood miRNA might be a promising and novel tool in the clinical diagnosis and treatment of PDAC.

## Figures and Tables

**Table 1:** MicroRNAs for the early detection of pancreatic cancer.

MicroRNAs	Function	Expression	Targets	Reference
miR-21	Oncogenic	↑	PI3K/AKT/PTEN, PDCD4, and Bcl-2/FasL	[16–20]
	Suppresses apoptosis and promotes proliferation			
miR-196a	Oncogenic	↑	—	[20]
miR-34	Tumor suppressor	↓	Bcl-2/Notch	[21, 22]
	Inhibits proliferation, invasion, and induces apoptosis			
miR-200	Tumor suppressor	↓	Notch, E-cadherin, and ZEB	[23]
Let-7	Tumor suppressor	↓	N-cadherin/ZEB1	[23]
	Reverses EMT and inhibits invasion			
miR-221	Oncogenic	↑	PI3K/AKT/PTEN, MMP-2, and MMP-9	[19, 24]
	Cancer cell invasion			
miR-145	Tumor suppressor	↓	KRAS	[25]
	Inhibits tumor growth			
miR-155	Oncogenic	↑	TP53INP	[26]
miR-15a	Tumor suppressor	↓	WANT3A, FGF7, and BMI-1	[27]
	Inhibits proliferation and EMT			
miR-506	Tumor suppressor	↓	SPHK1, PI3M	[28, 29]
	Inhibits proliferation and induces apoptosis			
miR-96	Tumor suppressor	↓	KRAS, AKT	[30]
	Inhibits proliferation and invasion and induces apoptosis			
miR-29a	Oncogenic	↑	Wnt/*β*-catenin	[31]

**Table 2:** Exosomal microRNA in pancreatic cancer patients.

Study	Exosomal microRNAs	Sample type	Level	Experiment/control group	Reference
Xu et al. (2011)	miR-196a	Plasma	↑	15/15	[41]
	miR-1246				
Madhavan et al. (2015)	miR-1246	Serum	↑	131/89	[40]
	miR-4644				
	miR-3976				
	miR-4306				
Chen et al. (2017)	miR-23b-3p	Plasma	↑	16/38	[42]
Lai et al. (2017)	miR-10b	Plasma	↑	29/17	[34]
	miR-21		↑		
	miR-30c		↑		
	miR-181a		↑		
	miR-let7a		↓		
Goto et al. (2018)	miR-191	Plasma	↑	61/22	[43]
	miR-21				
	miR-451a				
Takahasi et al. (2018)	miR-451a	Plasma	↑	6/50	[44]
